# Attenuated Vasodilator Effectiveness of Protease-Activated Receptor 2 Agonist in Heterozygous *par2* Knockout Mice

**DOI:** 10.1371/journal.pone.0055965

**Published:** 2013-02-07

**Authors:** John C. Hennessey, John J. McGuire

**Affiliations:** Cardiovascular Research Group, Division of BioMedical Sciences, Faculty of Medicine, Memorial University of Newfoundland, St. John's, Newfoundland and Labrador, Canada; Universidade Federal do Rio de Janeiro, Brazil

## Abstract

Studies of homozygous PAR2 gene knockout mice have described a mix of phenotypic effects *in vitro* and *in vivo*. However, there have been few studies of PAR2 heterozygous (wild-type/knockout; PAR2-HET) mice. The phenotypes of many hemi and heterozygous transgenic mice have been described as intermediates between those of wild-type and knockout animals. In our study we aimed to determine the effects of intermediary *par2* gene zygosity on vascular tissue responses to PAR2 activation. Specifically, we compared the vasodilator effectiveness of the PAR2 activating peptide 2-furoyl-LIGRLO-amide in aortas of wild-type PAR2 homozygous (PAR2-WT) and PAR2-HET mice. In myographs under isometric tension conditions, isolated aortic rings were contracted by alpha 1-adrenoeceptor agonist (phenylephrine), and thromboxane receptor agonist (U46619) and then relaxation responses by the additions of 2-furoyl-LIGRLO-amide, acetylcholine, and nitroprusside were recorded. A Schild regression analysis of the inhibition by a PAR2 antagonist (GB-83) of PAR2 agonist-induced aortic ring relaxations was used to compare receptor expression in PAR2-WT to PAR2-HET. PAR2 mRNA in aortas was measured by quantitative real-time PCR. In aortas contracted by either phenylephrine or U46619, the maximum relaxations induced by 2-furoyl-LIGRLO-amide were less in PAR2-HET than in the gender-matched PAR2-WT. GB-83 was 3- to 4-fold more potent for inhibition of 2fly in PAR2-HET than in PAR2-WT. PAR2 mRNA content of aortas from PAR2-HET was not significantly different than in PAR2-WT. Acetylcholine- and nitroprusside-induced relaxations of aortas from PAR2-HET were not significantly different than in PAR2-WT and PAR2 knockout. An interesting secondary finding was that relaxations induced by agonists of PAR2 and muscarinic receptors were larger in females than in males. We conclude that the lower PAR2-mediated responses in PAR2-HET aortas are consistent with evidence of a lower quantity of functional receptor expression, despite the apparently normal PAR2 mRNA content in PAR2-HET aortas.

## Introduction

One of the most significant models developed to study the pharmacology of protease-activated receptor 2 (PAR2) is the *par2* gene knockout mouse (PAR2-KO). In the past fifteen years, researchers have created several PAR2-KO strains, which have been used to explore the role of PAR2 in various pathological conditions/models [1]. PAR2 activation is particularly interesting from the standpoint of new pharmaceutical development for treatment of vascular endothelium health. A considerable amount of literature has been published on the vascular actions of PAR2 [1], which include endothelium-dependent relaxation of vascular smooth muscle [2], and pro-inflammation activities [3]. In instances of cardiovascular disease where other endothelium-dependent vasodilators have an attenuated effectiveness, PAR2-mediated vasodilation is retained [4]–[7]. PAR2 can be activated by trypsin-like serine proteases [4], [8]–[10], and by PAR2-activating peptides e.g. 2-furoyl-LIGRLO-amide (2fly) [4]. Only in the recent years past have researchers published their findings about *in vivo* and *in vitro* effects of the non-peptide PAR2 antagonist GB-83 [11]. So far there is only limited phenotype descriptions about PAR2 null heterozygous mice (PAR2-HET), which have half of the *par2* gene content of wild-type PAR2 mice (PAR-WT).

In a study based on an experimental mouse model of arthritis, significantly higher measures of synovium and periarticular tissue inflammation were reported in PAR2-WT than in both PAR2-HET and PAR2-KO [12]. Though PAR2-HET showed moderate joint tissue damage as determined by their histological scores for arthritis, the joint tissue phenotype index was closer in scores to PAR2-WT than to PAR2-KO. Other studies have shown that phenotypes of heterozygous transgenic mice may correspond better to the phenotype of the wild-type than to the homozygous transgenic mice [13]. For example, heterozygous pancreatic beta cell dysfunction diabetic gene *db* mice do not have pancreatic abnormalities, and thus, were similar to wild-type mice [14]. Researchers propose that compensatory mechanisms allow the *db* heterozygotes to retain the apparent wild-type phenotype [14]. Another proposed explanation for the phenotype equivalency between heterozygotes and wild-types is the circumstance of tissue spare receptors; more receptors are expressed in the tissues than needed for maximal effect [15]. Clearly, the regulation of phenotype varies with transcript content, but the extent of phenotype change for different tissues is quite variable. The peculiarities of gene regulation and vascular phenotype can also be confounded by interaction with gender (e.g. transgenic NOS knockout mice [16] and muscarinic (M_3_) activation in rats [17]). Despite these observations, little is known about the general impact of gender on PAR2 vascular biology.

The main aim of our current study was to determine the effect of *par2* zygosity on PAR2 activity as assessed by the relaxation of vascular smooth muscle in PAR2-HET aortas and compared to PAR2-WT. The main experimental approach was to measure the isometric tension responses of aortas after exposure to different vasodilators (PAR2 agonist (2fly), acetylcholine, and nitroprusside). Based on evidence of a very small attenuation of PAR2-mediated relaxation in PAR2-HET versus PAR2-WT, we conducted myograph experiments with the PAR2 antagonist GB-83 that quantified the tissue spare receptors in aortas of PAR2-WT and PAR2-HET. Finally, PAR2 mRNA expression was measured in aortas by quantitative real-time PCR. In light of the potential interaction of gender with endothelium-mediated relaxation mechanisms, descriptive comparisons of the vascular pharmacology of aortas from PAR2-HET versus WT and KO, and males versus females were deemed convenient secondary objectives. The results indicate that the aortas of PAR2-HET were less responsive to activation of PAR2, and the expression of functional PAR2 in aortas was less in HET than in WT; despite constitutive levels of PAR2 mRNA not being different between HET and WT. These findings suggest a non-linear relationship between *par2* gene copy number and the pathophysiological consequences of PAR2 activation.

## Methods

### Ethics Statement

All animal handling and experimental procedures were approved by the Institutional Animal Care Committee of Memorial University in accordance with the guidelines and principles of the Canadian Council of Animal Care.

### Animals

Stock breeders of C57BL/6J (PAR2-WT) and B6.Cg-*F2rl*1*^tm1Mslb^*/J (PAR2-KO) mice were purchased from Jackson Laboratory (Bar Harbor, ME). Multiple PAR2-WT and PAR2-KO mice breeders were crossed to produce F_2_ (PAR2-HET) mice having different F_1_ lineages. These F_2_ PAR2-HET were crossed to produce F_3_ which were used to generate F_4_ offspring; both F_3_ and F_4_ were used in our experiments. Littermate mice were housed and separated by sex in air filter-topped cages within a specific pathogen-free barrier facility of the Health Sciences Centre at Memorial University. Mice were provided food and water *ad libitum*.

### Sources of drugs and materials for myograph studies

Unless stated otherwise, all drugs and reagents were obtained from Sigma Aldrich (Oakville, Ontario, Canada). 2-furoyl-leu-ile-gly-arg-leu-orn-amide (2-furoyl-LIGRLO-amide) was obtained from the University of Calgary Peptide Synthesis (Calgary, Alberta, Canada). PAR2 antagonist GB-83 was obtained from Axon Medchem (Groningen, Netherlands).

### Genotyping

**Figure 1:**
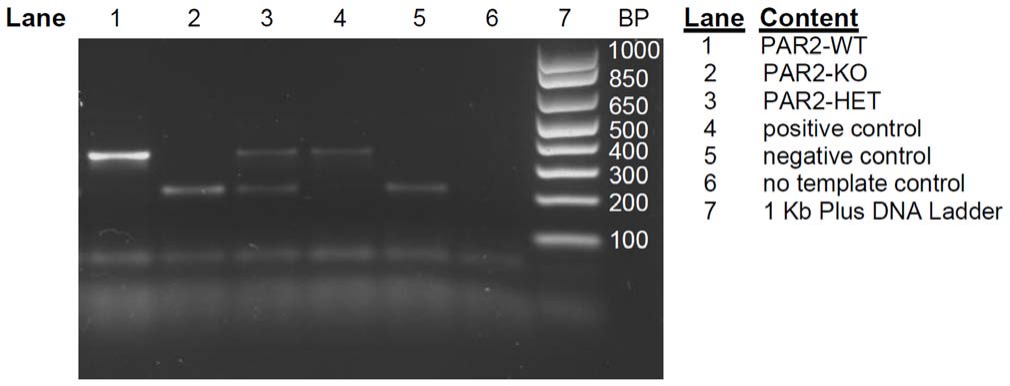
Representative *par*2 genotyping results of WT, HET and KO mice. PCR products from mouse tail samples were separated by agarose gel electrophoresis. *BP*; base pairs. Positive identification of *par*2 gene, 385 base pairs, in lanes: 1, 3 and 4. Positive identification for *neomycin* gene, 198 base pairs, in lanes: 2, 3 and 5. Previously identified PAR2-WT (lane 4) and PAR2-KO (lane 5) ran as positive and negative controls, respectively. Lane 7 contains the 1 Kb Plus DNA Ladder used for band identification.

Mice (21 days of age) were genotyped according to the supplier's protocol with minor modification (Jackson Laboratory, Bar Harbor, ME). Mouse tail clips (2 mm) were incubated in 50 mM NaOH at 95°C for 1 h then diluted with Tris HCl buffer (pH 7.5) and centrifuged at 10,000× *g* for 2 min before extracting supernatant containing the DNA. DNA samples were stored at −20°C until used in assays. DNA concentrations were determined by measuring *A*_260__nm_ on a NanoDrop 1000 spectrophotometer (Fischer Scientific, Ottawa, ON). PCR was carried out with three oligonucleotide primer sets to target the characteristic genes in PAR2-WT, PAR-HET and PAR2-KO [3]. The reaction mixture with primers amplified a portion of exon 2 in the *par*2 gene present in PAR2-WT and PAR2-HET and a fragment of the *neomycin* gene present in PAR2-KO, and PAR2-HET. PAR2-HET was identified as containing amplified *par2* exon 2 and *neomycin* gene fragments. Gels cast were 1.5% agarose containing SYBR Safe DNA chelating dye. A 1 kb DNA ladder (100 bp resolution) was run parallel to PCR products for discerning fragment sizes. Gels were run for 1.5 hours at 90 V to separate the DNA bands at a distance ≥4 cm from the wells. Migrated PCR products were imaged with Alpha Imager EP (Cell Biosciences, Santa Clara, CA) using trans-ultraviolet light detection. A sample genotyping gel for the above reaction is shown in Figure 1.

### Vascular reactivity

PAR2-WT, PAR2-HET and PAR2-KO (13 to 26 weeks of age) were euthanized by overdose inhalation of isoflurane followed by cardiac puncture. Descending thoracic aortas were removed from mice and kept in ice-cold Krebs buffer until cleaned of surrounding adipose and other adhering tissues. Krebs buffer (pH 7.4, 37°C) was bubbled continuously with 95% O_2_/5% CO_2_ and was comprised of 114 mM NaCl, 4.7 mM KCl, 0.8 mM KH_2_PO_4_, 1.2 mM MgCl_2_.6H_2_O, 2.5 mM CaCl_2_, 11 mM d-glucose, and 25 mM NaHCO_3_. Rings of aortas (1 to 2 mm lengths) were mounted on 200 µm diameter hooks in myograph chambers (DMT 610M, DMT620M; Danish Myograph Technologies, Aarhus, DK) under isometric tension conditions. Normalized resting tension was 13.3 kPa, which had been optimal for obtaining maximal relaxation responses by agonists in a pilot study by JCH. Tissue viability was determined by measuring aortic ring contractions to K^+^ (30 to 120 mM). The high K^+^-induced contractions were not different among *par2* genotypes, and genders (*P*>0.05). An aortic ring passed viability testing if these contractions were >1 mN/mm length of aorta. Contractility of aortas was measured by concentration-response curve (CRC) relationships to α_1_-adrenergic receptor agonist phenylephrine, and thromboxane receptor agonist U46619. Contracting agents were used to produce submaximal tension increases above resting tension levels prior to determining the relaxations by 2-furoyl-LIGRLO-amide, acetylcholine, and nitroprusside. In the experiments that assessed NOS inhibition, aortas were exposed to an effective single concentration of NO synthase (NOS) inhibitor (N_ω_-nitro L-arginine-methyl ester; L-NAME). In the experiments assessing PAR2 inhibition, aortic rings were incubated for 20 min with either vehicle (controls; DMSO 0.1% (v/v) water) or GB-83 (0.1 µM–60 µM) and then cumulative 2fly, acetylcholine, and nitroprusside concentration-relaxation response relationships were constructed in aortas, submaximally contracted by addition of phenylephrine (0.7 µM).

### Quantitative real-time PCR

Purified RNA from lengths of aortas (2 mm, 3–5 mg) was isolated using RNeasy Fibrous Kit QIAshredder spin columns according to manufacturer's directions (Qiagen, Mississauga, ON) and a previously validated approach [6]. RNA samples were stored at −20°C until used. RNA yield was determined using a NanoDrop 1000 spectrophotometer (Fischer Scientific, Ottawa, ON). Real-time PCR of the target and reference genes, *par*2 and *gapdh*, respectively, was conducted using RNA-to-C_T_ kit on ABI 7000 Real-time PCR System (Applied Biosystems, Streetsville, ON).

### Data Analyses

Myograph data reported in tables and the symbols on graphs are mean ± standard error of the mean (S.E.M.). *n* = number of mice. Graph Pad Prizm software ver. 4.1 was used to generate curves and analyse statsitics. E_max_ was the observed maximum effect for each drug. Drug CRC represent the best-fit relationship for data by nonlinear regression using a four parameter logistic equation, which was also used to calculate pD_2_, and hill slope variables for each aorta. Effect = Bottom+(E_max_ - Bottom)/(1+10 ^log EC50 - log [Drug]) * hill slope^); Bottom is an asymptote equal to 0; pD_2_ is negative logarithm base 10 of the EC_50_ value (drug concentration (M) resulting in half of maximum effectiveness); % relaxation = reversal of agonist-induced contraction i.e. 100% relaxation is complete reversal of vasoconstrictor-induced tone. Contractions are reported as effective pressure change (kPa), which were calculated by normalization of the recorded isometric tension changes relative to wall length and internal circumference of each aortic ring. For myograph data, statistical comparisons between two groups were made using Student's t-test for unpaired data, and more than two groups were made using two-way anova followed by Bonferroni post-hoc tests for multiple pairwise comparisons.

To compare the expression of functional spare receptors in PAR2-WT to PAR2-HET, a linear regression analyses was used: pD_2_ = slope * (log_10_[GB-83])+intercept; where pD_2_ is determined for the agonist (as described above) in the presence of varying concentrations of antagonist (GB-83). Slopes were compared to 0 by Student's t-test (*P*<0.05 was considered significant). Two data points in PAR2-WT were excluded because these were considered as being outliers of the linear regression scatter plots (dispersion outside the 95% confidence intervals for the slopes). Equilibrium binding constants for GB-83 (*k*_GB-83_) were calculated from 1/slope as determined by a regression of the Schild equation [18], [19] defined by the formula: (*r*_A_−1) = (1/*k*_GB-83_)[GB-83]+intercept; where *r*_A_ is defined as the ratio of agonist (2fly) EC_50_ in the presence of antagonist (GB-83) to agonist EC_50_ in the absence of inhibitor. For mRNA expression data, statistical comparisons were made using REST software as previously described [6]. *P*<0.05 was considered significant. Gene expression data are expressed as a box-and-whisker plot with error bars representing the upper and lower 95% confidence intervals.

## Results

### Effect of par2 zygosity and gender on PAR2-mediated vasodilation

**Figure 2:**
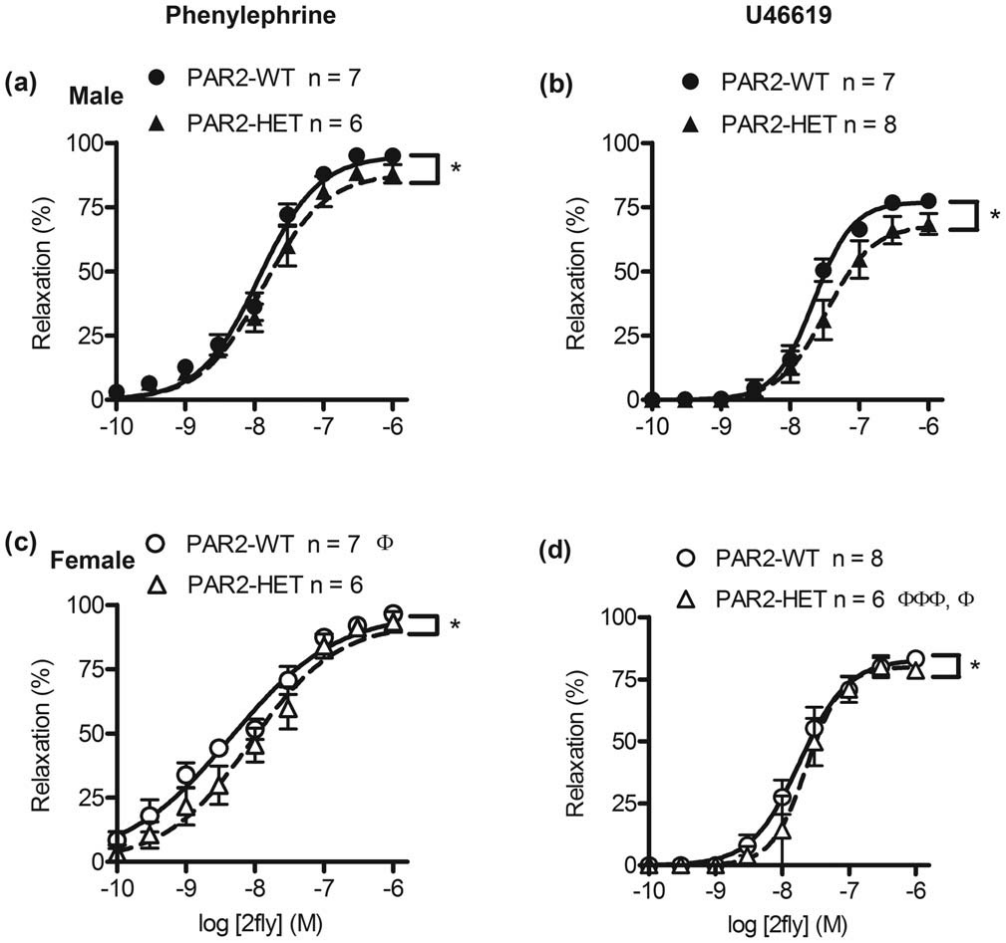
Relaxation effects of 2-furoyl-LIGRLO-amide (2fly) on untreated α_1_ receptor agonist and thromboxane A_2_ receptor agonist-contracted mouse aortas across PAR2 genotypes. Male (top) and female (bottom) aortic rings were contracted submaximally by phenylephrine (a, c) and U46619 (b, d) then relaxed by the cumulative addition of 2-furoyl-LIGRLO-amide (2fly) under isometric tension conditions. Symbols are means ± S.E.M., n = number of mice. Lines represent 4 parameter logistic curves which calculate the variables: pD_2_, E_max_ and hill-slope. Variables were compared by 2 way ANOVA (sex x genotype) followed by Bonferonni post-hoc tests. (a, b, c, d)*P<0.05, E_max_, genotype effect. (c) ^Φ^ *P*<0.05, pD_2_ and hill-slope, PAR2-WT male vs female. (d) ^ΦΦΦ^ *P*<0.001, hill-slope, ^Φ^ *P*<0.05, E_max_, PAR2-HET male vs female.

To determine whether *par*2 zygosity affected PAR2-mediated vasodilation, aortas from PAR2-WT, PAR2-HET and PAR2-KO were contracted submaximally by phenylephrine and then exposed to 2fly. In phenylephrine-contracted aortas, the maximal effectiveness of 2fly was attenuated in PAR2-HET by 6% in males and 4% in females compared to PAR2-WT (*P*<0.05, E_max_, Figure 2 *a, c* and Table 1). In males, the sensitivity to 2fly was not different between PAR2-WT and PAR2-HET (*P*>0.05, pD_2_ and hill slope, Figure 2 *a* and Table 1). However, in phenylephrine-contracted aortas from female PAR2-HET there was a decrease in sensitivity to 2fly at the lower range of concentrations (from 0.1 nM to 30 nM) versus PAR2-WT (*P*<0.05, pD_2_ and hill slope, Figure 2 *c* and Table 1). To determine whether the selection of contractile agonist affected these results, 2fly-induced relaxation was also measured in aortas that were contracted submaximally by U46619. The maximal effectiveness of 2fly was also attenuated in PAR2-HET by 9% in males and 5% in females compared to PAR2-WT (*P*<0.05, E_max_, Figure 2 *b, d* and Table 2). In female PAR2-HET, the 2fly CRC also indicated an increase in steepness by 2 fold and increase in relaxation by 11% (*P*<0.001, hill slope, *P*<0.05 E_max_, Figure 2 *d* and Table 2) compared to male PAR2-HET. As we had expected, 2fly did not relax either the phenylephrine (Table 1) or U46619 (Table 2)-contracted aortas from PAR2-KO (Tables 1 and 2).

**Table 1:** Phenylephrine-contracted 2-furoyl-LIGRLO-amide (2fly), acetylcholine and nitroprusside concentration-relaxation relationships for PAR2-WT, PAR2-HET, and PAR2-KO mouse aortas.

Vasodilator	Genotype	Sex	n	pD_2_ a	E_max_ (%)b, c	Hill slope^f^
2fly	PAR2-WT	m	7	8.0±0.1	95±2	1.2±0.2
		f	7	8.4±0.1^f^	96±1	0.6±0.1^f^
	PAR2-HET	m	6	7.8±0.1	89±3^b^	1.1±0.1
		f	6	8.1±0.2	92±3^b^	0.8±0.1
	PAR2-KO	m	7	0*	0*	0*
		f	6	0*	0*	0*
Acetylcholine	PAR2-WT	m	8	7.7±0.1	83±4	1.0±0.1
		f	7	8.4±0.1^d^	93±2^c^	0.7±0.1
	PAR2-HET	m	5	7.5±0.2^a^	84±3	0.8±0.1
		f	6	8.2±0.1^a^^, ^ e	91±2^c^	0.7±0.1
	PAR2-KO	m	7	8.1±0.1^a^	90±4	0.8±0.1
		f	7	8.4±0.1^a^	90±1^c^	0.8±0.1
Nitroprusside	PAR2-WT	m	8	8.6±0.1	98±1	1.0±0.1
		f	7	8.7±0.1	92±3^c^	0.9±0.1
	PAR2-HET	m	6	8.6±0.1	97±1	1.1±0.1
		f	6	8.7±0.1	94±3^c^	0.9±0.1
	PAR2-KO	m	7	8.7±0.1	97±1	0.8±0.1
		f	7	8.9±0.1	96±2^c^	0.8±0.1

Values are means ± S.E.M., n = number of mice/group. Variables were determined by curve fitting vasodilator-induced relaxation responses to a 4 parameter logistic curve. Data were analyzed by 2 way ANOVA (sex x genotype) followed by Bonferonni post-hoc testing. n/d, not determined. 0*, data not significantly different from zero, *P*>0.05.

a *P*<0.01,

b *P*<0.05, main genotype effect.

c *P*<0.05, main gender effect.

d *P*<0.001,

e *P*<0.01,

f *P*<0.5, male vs female.

**Table 2:** U46619-contracted 2-furoyl-LIGRLO-amide (2fly), acetylcholine and nitroprusside concentration-relaxation relationships for PAR2-WT, PAR2-HET, and PAR2-KO mouse aortas.

Vasodilator	Genotype	Sex	n	pD_2_	E_max_ (%)^b^^, ^ c	Hill slope^a^
2fly	PAR2-WT	m	7	7.6±0.1	79±2	1.9±0.3
		f	8	7.7±0.1	86±2	1.4±0.1
	PAR2-HET	m	8	7.4±0.1	70±4^b^	1.5±0.1
		f	6	7.6±0.1	81±4^b^^, ^ e	3.2±0.5^d^
	PAR2-KO	m	7	0*	0*	0*
		f	8	0*	0*	0*
Acetylcholine	PAR2-WT	m	7	7.4±0.2	62±3^c^	1.3±0.2
		f	7	7.6±0.1	79±5^c^^, ^ e	1.3±0.2
	PAR2-HET	m	7	7.3±0.2	61±7^c^	1.4±0.3
		f	7	7.7±0.2	79±3^c^^, ^ e	1.0±0.2
	PAR2-KO	m	8	7.6±0.1	87±1^c^^, ^ f	0.9±0.1
		f	7	7.8±0.3	79±3^c^	0.9±0.2
Nitroprusside	PAR2-WT	m	6	7.9±0.2	90±1	0.8±0.1
		f	8	8.3±0.3	85±3	2.0±0.6^a^
	PAR2-HET	m	6	8.0±0.2	88±2	1.0±0.1
		f	7	7.7±0.2	83±2	2.9±0.9^a^
	PAR2-KO	m	6	8.2±0.2	81±3	1.2±0.2
		f	7	8.1±0.1	84±3	2.3±0.7^a^

Values are means ± S.E.M., n = number of mice/group. Variables were determined by curve fitting vasodilator-induced relaxation responses to a 4 parameter logistic curve. Data were analyzed by 2 way ANOVA (sex x genotype) followed by Bonferonni post-hoc testing. n/d, not determined. 0*, data not significantly different from zero, *P*>0.05.

a *P*<0.01, main gender effect.

b *P*<0.05, main genotype effect.

c *P*<0.01, main interaction effect.

d *P*<0.001,

e *P*<0.05, male vs female.

f *P*<0.001, PAR2-WT vs PAR2-KO.

### Effect of par2 zygosity and gender on NOS-mediated vasodilation

**Figure 3:**
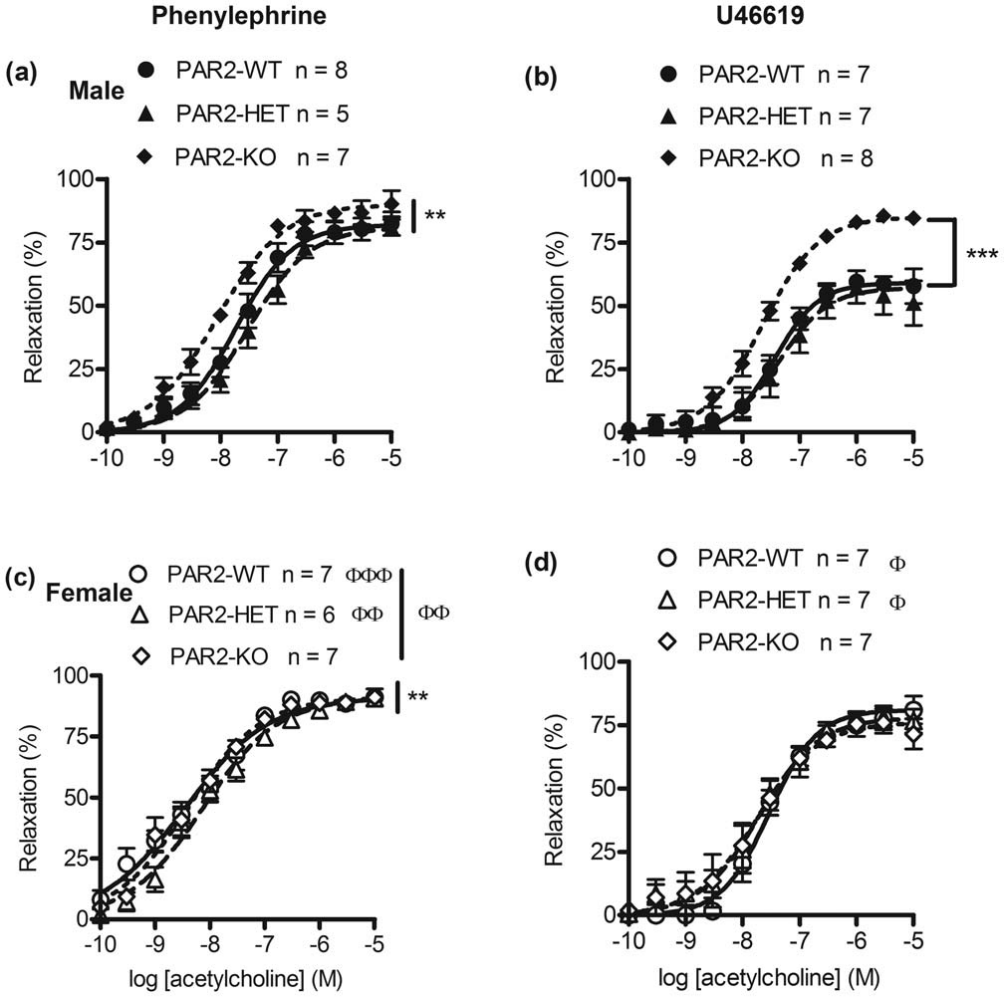
Relaxation effects of acetylcholine on untreated α_1_ receptor agonist and thromboxane A_2_ receptor agonist-contracted mouse aortas across PAR2 genotypes. Male (top) and female (bottom) aortic rings were contracted submaximally by phenylephrine (a, c) and U46619 (b, d) then relaxed by the cumulative addition of acetylcholine under isometric tension conditions. Symbols are means ± S.E.M., n = number of mice. Lines represent 4 parameter logistic curves which calculate the variables: pD_2_, E_max_ and hill-slope. Variables were compared by 2 way ANOVA (sex x genotype) followed by Bonferonni post-hoc tests. (a, c) ^**^ *P*<0.01, pD_2_, main genotype effect. (b) ^***^ *P*<0.001, E_max_, PAR2-WT vs PAR2-KO genotype effect. (c) ^Φ^ *P*<0.05, E_max_, main gender effect. ^ΦΦ^ *P*<0.05, pD_2_, PAR2-HET male vs female. ^ΦΦΦ^ *P*<0.001, pD_2_, PAR2-WT male vs female. (d) ^Φ^ *P*<0.05, E_max_, PAR_2_-WT and PAR2-HET male vs female.

To determine whether *par*2 zygosity affected NOS-mediated vasodilation, aortas were contracted submaximally by phenylephrine then exposed to acetylcholine. Relaxations by acetylcholine were not significantly different between PAR2-WT, PAR2-HET and PAR2-KO (*P*>0.05, all variables, Figure 3 *a, c* and Table 1). However, acetylcholine was more effective and five times more potent in aortas from female PAR2-WT, and PAR2-HET than male PAR2-WT, and PAR2-HET (Figure 3 *c* and Table 1). However, to determine whether the choice of vasoconstrictor affected these results, relaxations by acetylcholine were also recorded in aortas that were contracted submaximally by U46619. The maximum relaxation by acetylcholine was higher in aortas from male PAR2-KO by 25% compared to male PAR2-WT (*P*<0.001, E_max_, Figure 3 *b* and Table 2). In addition male PAR2-WT and PAR2-HET had attenuated relaxation by 17% and 18% respectively when compared to females (*P*<0.05, E_max_, Figure 3 *b* and Table 2). Otherwise there were no significant differences in acetylcholine CRC between PAR2-WT, PAR2-HET and PAR2-KO.

### Effect of par2 zygosity and gender on nitroprusside-induced vasodilation

**Figure 4:**
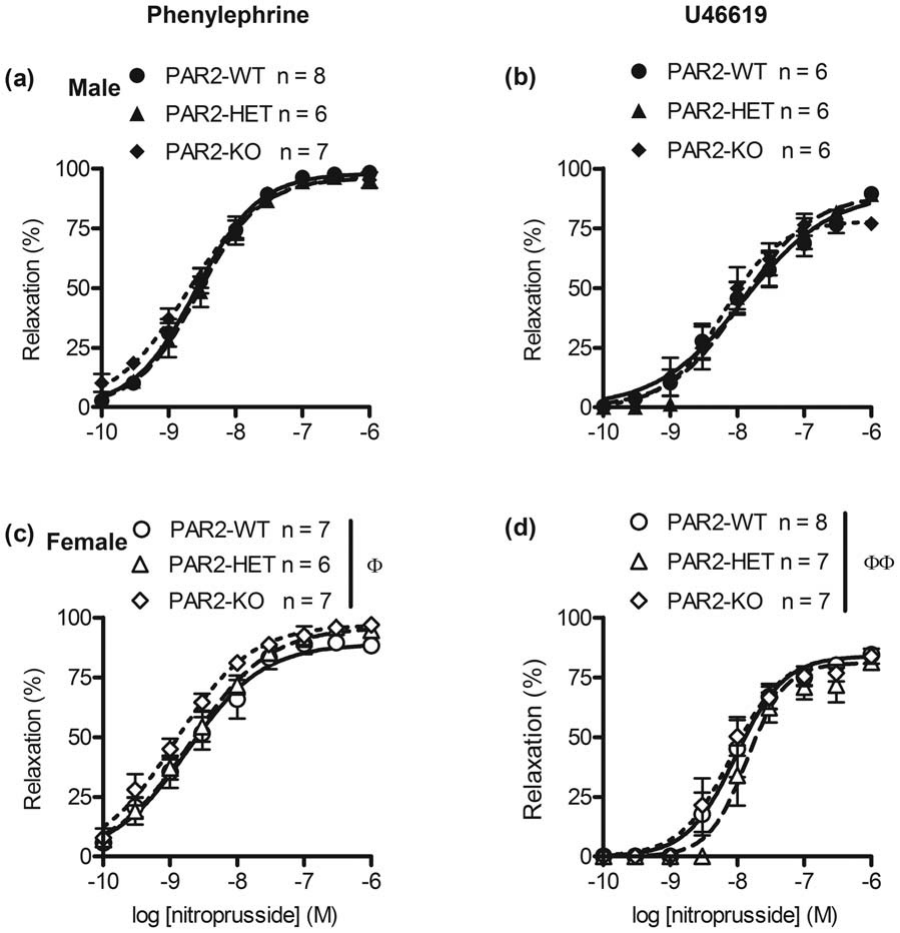
Relaxation effects of nitroprusside on untreated α_1_ receptor agonist and thromboxane A_2_ receptor agonist-contracted mouse aortas across PAR2 genotypes. Male (top) and female (bottom) aortic rings were contracted submaximally by phenylephrine (a, c) and U46619 (b, d) then relaxed by the cumulative addition of nitroprusside under isometric tension conditions. Symbols are means ± S.E.M., n = number of mice. Lines represent 4 parameter logistic curves which calculate the variables: pD_2_, E_max_ and hill-slope. Variables were compared by 2 way ANOVA (sex x genotype) followed by Bonferonni post-hoc tests. (c) ^Φ^ *P*<0.05, E_max_, main gender effect. (b) ^ΦΦ^ *P*<0.01, hill-slope, main gender effect.

To determine whether *par*2 zygosity affected vascular smooth muscle's sensitivity to NO, aortas were contracted submaximally by phenylephrine and then exposed to nitroprusside. There were no significant differences within gender of PAR2-WT, PAR2-HET, and PAR2-KO. However, maximum relaxations by nitroprusside were measurably larger in aortas from males vs. females (*P*<0.05,E_max_, Figure 4 *c* and Table 1). Conversely we determined that the choice of vasoconstrictor affected these results when relaxations by nitroprusside were recorded in aortas that were contracted submaximally by U46619. There were no significant differences between male PAR2-WT, PAR2-HET and PAR2-KO. Also, there were no significant differences between female PAR2-WT, PAR2-HET and PAR2-KO. However, nitroprusside CRC in aortas from females were approximately 2-times steeper than male PAR2-WT, PAR2-HET and PAR2-KO (*P*<0.05, hill slope, Figure 4 *d* and Table 2).

### Effects of par2 zygosity and gender on contractions of aortas by phenylephrine and U46619

**Figure 5:**
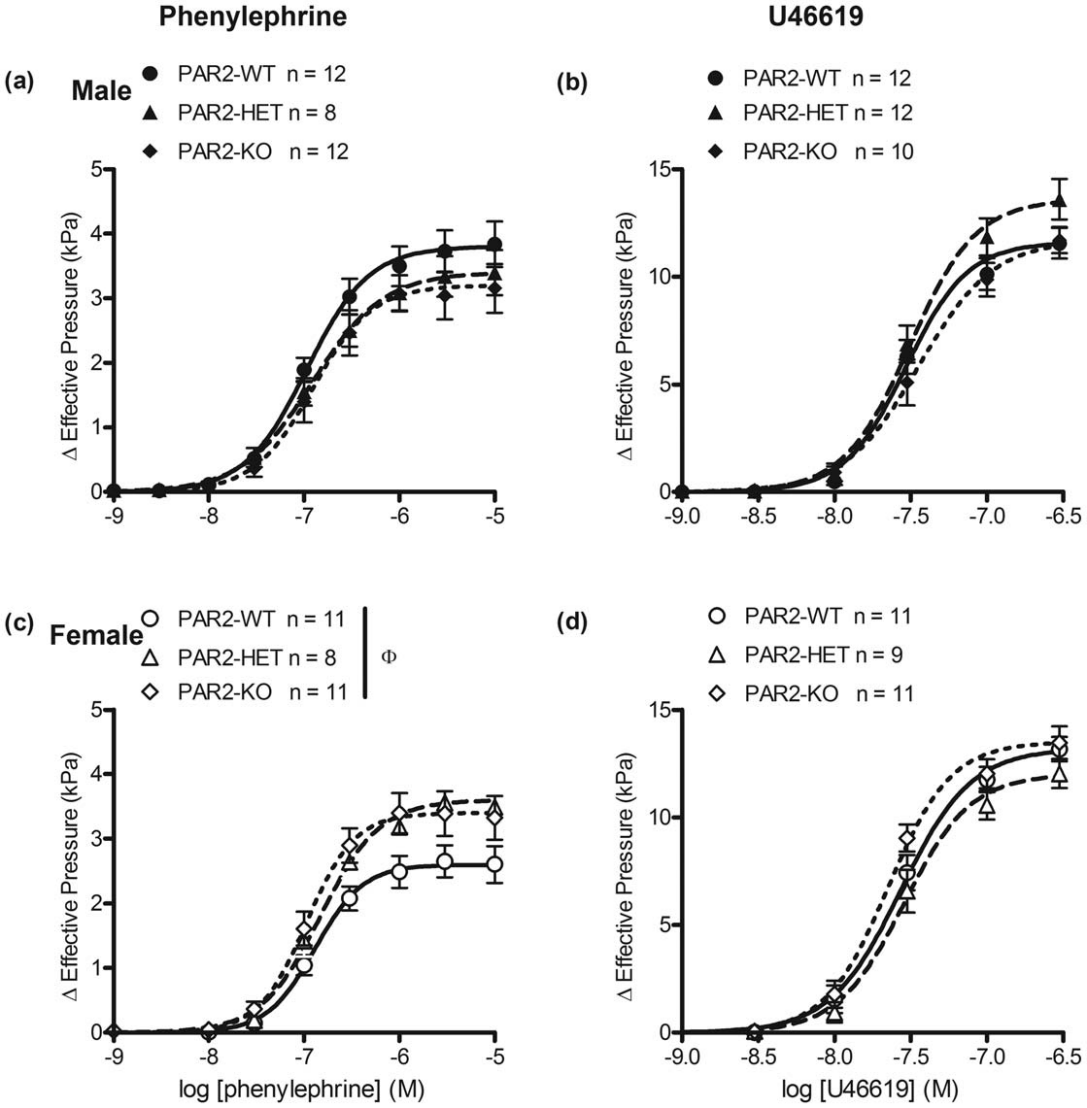
Contractile effects of an α_1_ adrenergic receptor agonist and thromboxane A_2_ receptor agonist on untreated mouse aortas across PAR2 genotypes. Male (top) and female (bottom) aortic rings were contracted by the cumulative addition of phenylephrine (a, c) and U46619 (b, d) under isometric tension conditions. Symbols are means ± S.E.M., n = number of mice. Lines represent 4 parameter logistic curves which calculate the variables: pD_2_, E_max_ and hill-slope. Variables were compared by 2 way ANOVA (sex x genotype). (c) ^Φ^ *P*<0.05, hill-slope, main gender effect.

To determine whether *par*2 zygosity affected α_1_-adrenergic receptor agonist-induced vascular reactivity, we measured the contractions of aortic rings by cumulative concentrations of phenylephrine. Phenylephrine-induced contractions were not different between PAR2-WT, PAR2-KO and PAR2-HET (*P*>0.05 for all variables, Figure 5 *a, c* and Table 3). Interestingly, phenylephrine CRCs in the untreated aortas were characterized by an increased steepness of these relationships in female vs. male mice (*P*<0.05, hill slope, Figure 5 *c* and Table 3). To determine the effect of *par*2 gene on thromboxane A_2_ agonist-induced vascular reactivity, we measured the contractions of aortic rings by U46619. U46619 CRCs were not different in aortas from PAR2-WT, PAR2-KO, and PAR2 HET. Gender did not affect U46619-induced contractions of aortas (*P*>0.05, all variables, Figure 5 *b, d* and Table 3).

**Table 3:** Phenylephrine and U46619 concentration-contraction relationships for PAR2-WT, PAR2-HET, and PAR2-KO mouse aortas.

Genotype	Sex	n	pD_2_	E_max_ (kPa)	Hill slope^a^
Phenylephrine					
PAR2-WT	m	12	7.0±0.1	4.0±0.4	1.4±0.1
	f	11	6.9±0.1	2.7±0.3	1.8±0.1^a^
PAR2-HET	m	8	6.9±0.1	3.5±0.3	1.2±0.1
	f	8	6.8±0.1	3.7±0.3	1.5±0.1^a^
PAR2-KO	m	12	6.9±0.1	3.3±0.3	1.6±0.3
	f	11	6.9±0.1	3.6±0.3	1.7±0.2^a^
U46619					
PAR2-WT	m	12	7.5±0.1	11.6±0.7	2.4±0.2
	f	11	7.6±0.1	13.2±0.6	2.3±0.2
PAR2-HET	m	12	7.5±0.1	13.8±1.0	2.4±0.3
	f	9	7.5±0.1	12.0±0.7	2.4±0.2
PAR2-KO	m	10	7.4±0.1	11.7±0.6	2.6±0.3
	f	11	7.7±0.1	13.5±0.8	2.5±0.2

Values are means ± S.E.M., n = number of mice/group. Variables were determined by curve fitting phenylephrine and U46619-induced contraction responses to a 4 parameter logistic curve. Data were analyzed by 2 way ANOVA (sex x genotype) followed by Bonferonni post-hoc testing.

a *P*<0.05, main gender effect.

### Effect of NOS inhibitor l-NAME on phenylephrine and U46619-induced contractions

**Figure 6:**
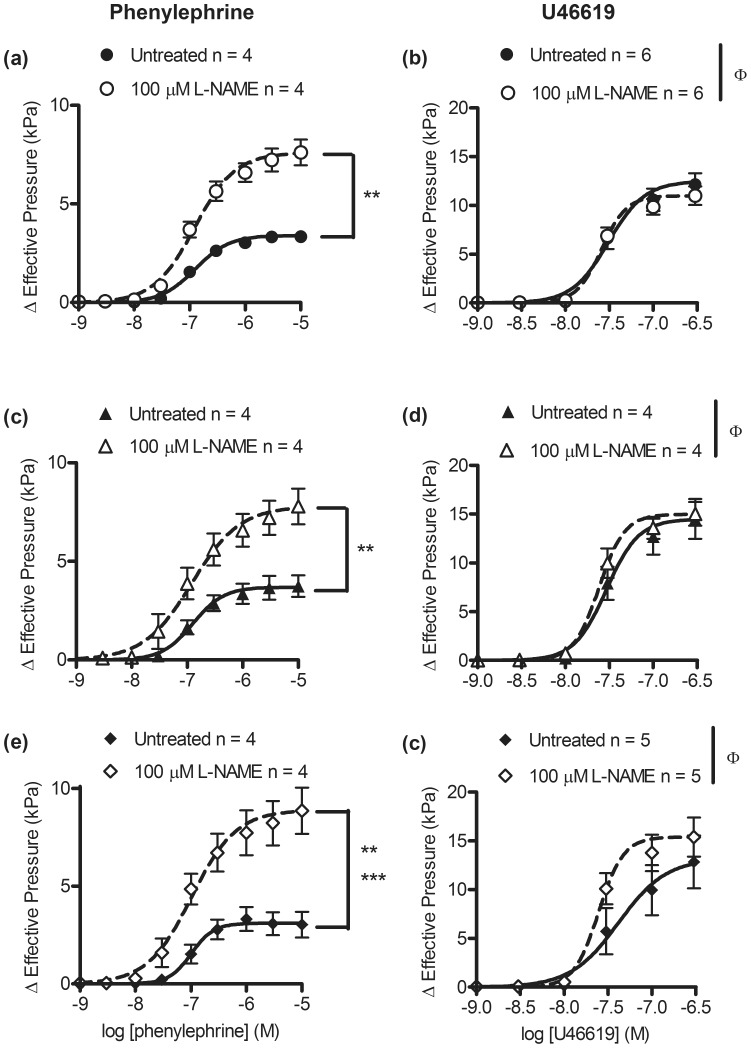
Effect of a nitric oxide synthase inhibitor, L-NAME, on α_1_ adrenergic receptor agonist and thromboxane A_2_ receptor-induced contractions of mouse aortas across PAR2 genotypes. PAR2-WT (a, b), PAR2-HET (c, d) and PAR2-KO (e, f) aortic rings were treated with 100 µM L-NAME and contracted by the cumulative addition of phenylephrine or U46619 under isometric tension conditions. Symbols are means ± S.E.M., n = number of mice. Lines represent 4 parameter logistic curves which calculate the variables: pD_2_, E_max_ and hill-slope. Variables were compared by 2 way ANOVA (treatment x genotype) followed by Bonferonni post-hoc tests. (a, c) **P<0.01, (e) ***P<0.001, E_max_, L-NAME vs untreated. (e)**P<0.01, hill-slope, L-NAME vs untreated. (b, d, f) ^Φ^ *P*<0.05, pD_2_, main treatment effect.

To determine whether *par*2 zygosity affected constitutive NOS activity in aortas, CRC for phenylephrine and U46619 were measured in the absence and presence of 100 µM l-NAME. In aortas from PAR2-WT, PAR2-HET, and PAR2-KO, treatment with l-NAME increased the phenylephrine elicited maximum contractions by ∼2-times vs. untreated aortas (*P*<0.05, E_max_, Figure 6 *a, c, e* and Table 4). In aortas from PAR2-WT, PAR2-HET and PAR2-KO, L-NAME treatment increased the sensitivity to U46619 by less than 2-fold compared to untreated aortas (*P*<0.05, pD_2_, Figure 6 *b, d, f* and Table 4).

**Table 4:** Phenylephrine and U46619 concentration-contraction response relationships for PAR2-WT, PAR2-HET and PAR2-KO mouse aortas in the presence and absence of L-NAME.

Genotype	Sex	n	Treatment	pD_2_ a	E_max_ (kPa)	Hill slope
Phenylephrine						
PAR2-WT	p	4	untreated	6.9±0.1	3.4±0.1	1.5±0.1
	p	4	L-NAME	6.9±0.1	7.6±0.7^b^	1.2±0.1
PAR2-HET	p	4	untreated	6.9±0.1	3.8±0.6	1.5±0.2
	p	4	L-NAME	6.9±0.1	7.8±0.9^b^	1.1±0.1
PAR2-KO	p	4	untreated	6.9±0.1	3.5±0.7	2.1±0.4
	p	4	L-NAME	7.0±0.1	8.9±1.2^c^	1.1±0.1^b^
U46619						
PAR2-WT	p	6	untreated	7.5±0.1	12.2±1.2	2.7±0.3
	p	6	L-NAME	7.6±0.1^a^	11.0±1.0	3.2±0.3
PAR2-HET	p	4	untreated	7.5±0.1	14.5±2.0	2.7±0.5
	p	4	L-NAME	7.6±0.1^a^	15.0±1.2	3.0±0.5
PAR2-KO	p	5	untreated	7.4±0.1	12.8±2.7	3.0±0.6
	p	5	L-NAME	7.6±0.1^a^	15.4±2.0	3.1±0.4

Values are means ± S.E.M., n = number of mice/group. Variables were determined by curve fitting phenylephrine and U46619-induced contraction responses to a 4 parameter logistic curve. Data were analyzed by 2 way ANOVA (treatment x genotype) followed by Bonferonni post-hoc testing. Sex (p) are pooled male and female groups.

a *P*<0.05, main treatment effect.

b *P*<0.01,

c *P*<0.001, within strain L-NAME vs untreated.

### Mechanism of PAR2-mediated vasodilation in PAR2-WT and PAR2-HET aortas

**Figure 7:**
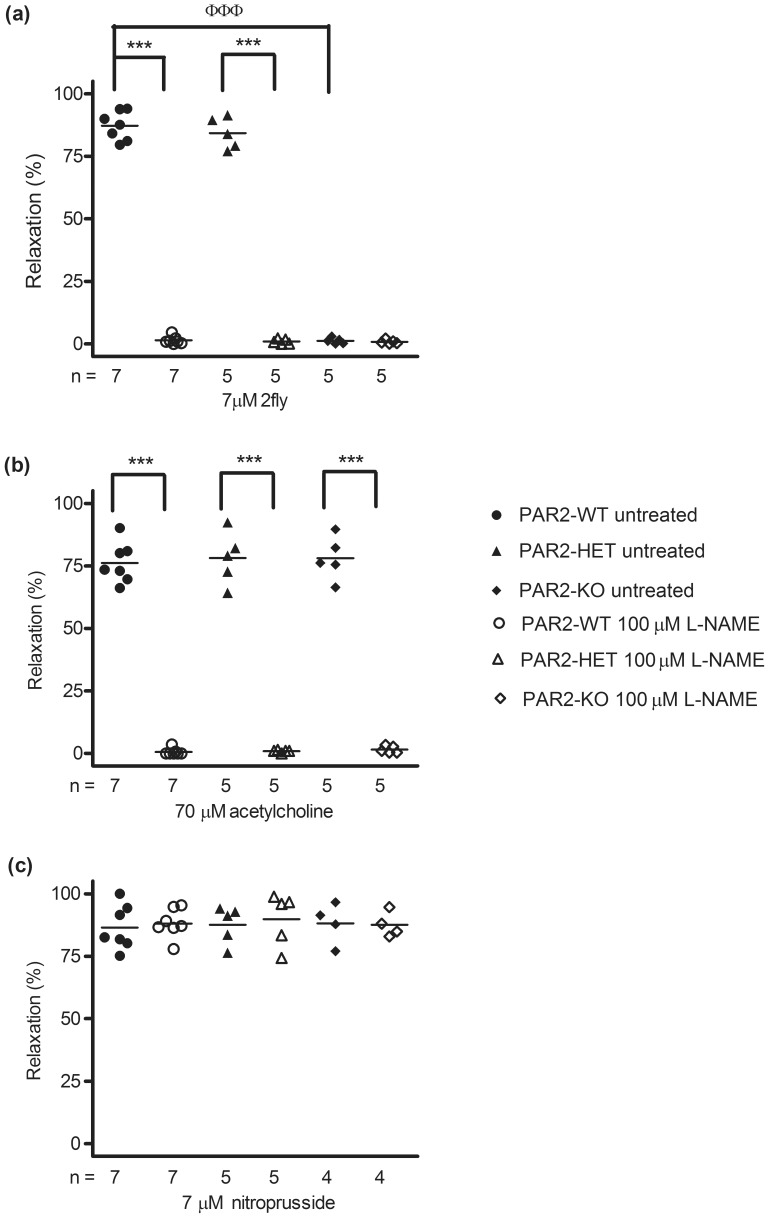
Effects of a nitric oxide synthase inhibitor, L-NAME, on acetylcholine, 2-furoyl-LIGRLO-amide (2fly) and nitroprusside-mediated relaxations in thromboxane A_2_ receptor agonist-contracted mouse aortas across PAR2 genotypes. Aortic rings were treated with 100 µM L-NAME then contracted submaximally by U46619 before single concentration addition of (a) 2-furoyl-LIGRLO-amide (2fly), (b) acetylcholine and (c) nitroprusside under isometric tension conditions. Symbols are mean E_max_ value, n = number of mice. Variables were compared by 2 way ANOVA (treatment x genotype) followed by Bonferonni post-hoc tests. (a)***P<0.001, E_max_, PAR2-WT and PAR2-HET L-NAME vs untreated. ^ΦΦΦ^ *P*<0.001, E_max_, untreated PAR2-WT vs untreated PAR2-KO. (b)***P<0.001, E_max_, L-NAME vs untreated.

To confirm that NOS alone is the main contributor to PAR2-mediated vasodilations, aortas were contracted submaximally by U46619 in the absence or presence of l-NAME and then exposed to a single maximal effective concentration of 2fly (0.7 µM). In the presence of L-NAME, 2fly-induced relaxations of aortas from PAR2-WT and PAR2-HET were abolished (Figure 7 *a*). As well, l-NAME abolished acetylcholine-induced relaxations, but not nitroprusside-induced relaxations in PAR2-WT, PAR2-HET and PAR2-KO (Figure 7 *b, c*). Equivalent results were obtained in aortas contracted submaximally by phenylephrine in the absence and presence of l-NAME (data not shown).

### Differential inhibition by PAR2 antagonist GB-83 of the relaxations of aortas by PAR2 agonist in PAR2-WT and PAR2-HET

**Figure 8:**
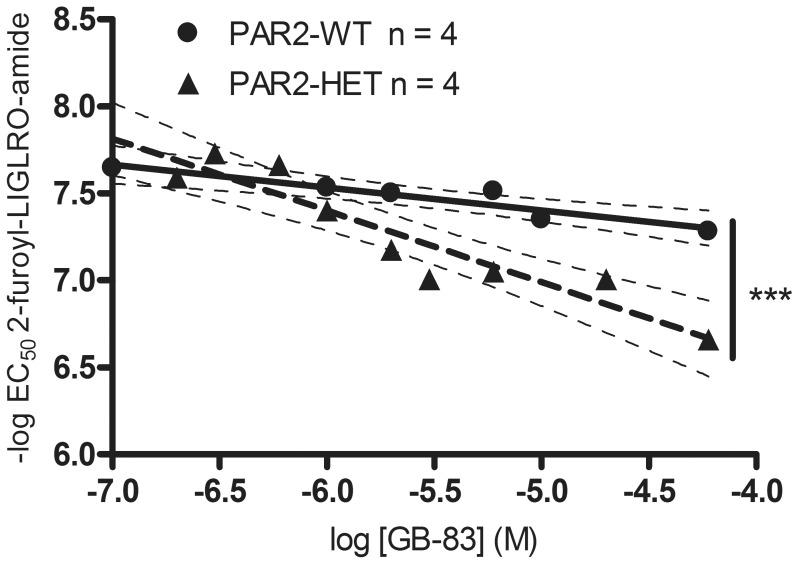
Differential inhibition by protease-activated receptor 2 antagonist GB-83 of 2-furoyl-LIGRLO-amide (2fly)-mediated relaxations of aortas from male PAR2-WT and PAR2-HET. Aortic rings were incubated for 20 min with either vehicle (controls; DMSO 0.1% (v/v) water) or GB-83 (0.1 µM–60 µM) and then cumulative 2fly concentration-relaxation response relationships were constructed in aortas, submaximally contracted by addition of phenylephrine (0.7 µM). Thick lines indicate simple linear regression analyses of 2fly EC_50_ values (symbols; log transformed) versus concentration of antagonist GB-83 (log transformed); thin dashed lines indicate S.E.M for each regression line. n = number of mice. ***P<0.001, slope regression coefficient of PAR-WT compared to PAR2-HET by Student's t-test. Coefficient of determination (R^2^) was 0.88 in both strains; *P<0.05, slopes for PAR2-WT and PAR2-HET compared to 0, and WT vs. HET. 2fly -log EC_50_ values of controls were 7.25±0.05 in PAR2-WT and 7.43±0.02 in PAR2-HET.

To determine whether a lower number of functional spare receptors in PAR2-HET may have accounted for the lower level of PAR2-agonist elicited activity in PAR2-HET aortas, we assessed the effect of PAR2 antagonist GB-83 in PAR2-WT and PAR2-HET. Exposure of aortas to GB-83 decreased the sensitivity of 2fly in both PAR2-WT and PAR2-HET (Figure 8). The inhibitory effects of equivalent doses of GB-83 were larger in PAR2-HET than in PAR2-WT (slopes for PAR2-HET vs. PAR2-WT, −0.41 vs. −0.13, *P*<0.001, Figure 8). Based on a Schild regression of the data in Figure 8, the equilibrium constants for GB-83 (*k*_GB-83_) were estimated at 53±17 µM in PAR2-WT and 14±2 µM in PAR-HET (*P*<0.01), which suggests a 3- to 4-fold difference in functional receptors between strains. There was no significant effect of GB-83 on phenylephrine-induced contractions, and acetylcholine-, and nitroprusside-mediated relaxations of aortas (data not shown).

### Aorta par2 gene expression in PAR2-HET mice

**Figure 9:**
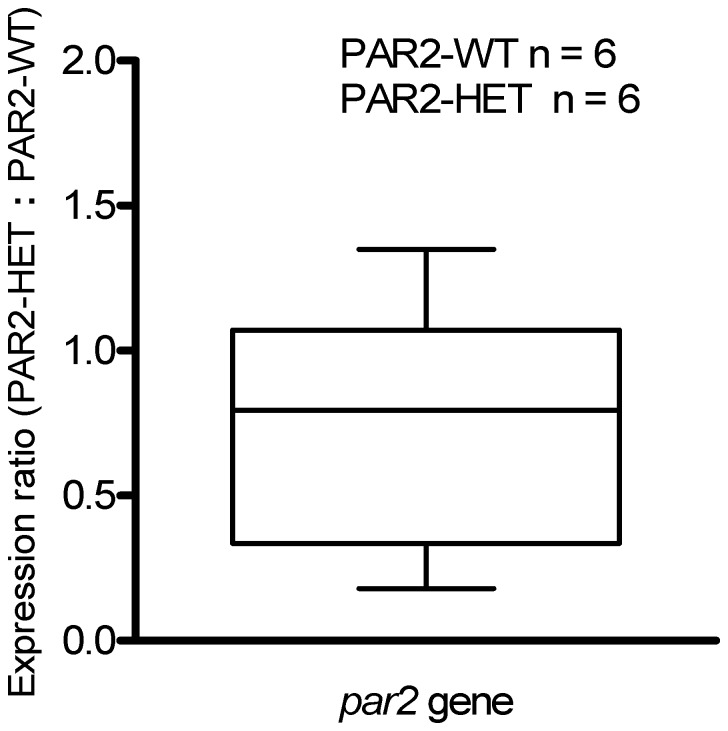
*par*2 mRNA expression (PAR2-HET relative to PAR2-WT) in mouse aortas. *par*2 mRNA from mouse aorta preparations was measured by real-time PCR. *par2* gene threshold cycle (C_T_) values were normalized to *gapdh* gene C_T_ values. Bars represent the 95% C.I. *par2* PCR reaction efficiency: 0.9517, *gapdh* PCR reaction efficiency: 0.9574. Expression ratio of *par*2 gene in PAR2-HET∶PAR2-WT is not significantly different from unity, *P*>0.05.

To determine whether *par2* gene expression was lowered in aortas of PAR2-HET vs. PAR2-WT, mRNA content was measured using quantitative real-time PCR. The *par2* mRNA level (normalized to *gapdh* expression within groups) in PAR2-HET was not different that in PAR2-WT (Figure 9). Under the same assay conditions no PAR2 mRNA was detected in PAR2-KO.

## Discussion

The main finding of our study was that the maximal effectiveness of the PAR2 agonist 2fly was attenuated in PAR2-HET compared to PAR2-WT. This finding was consistent with evidence obtained with the PAR2 antagonist GB-83 that indicated a lower number of functional spare receptors in aortas of PAR2-HET than in PAR2-WT. Despite the differential functional effects of both a PAR2 agonist and PAR2 antagonist in PAR2-WT and PAR2-HET, the content of PAR2 mRNA was not found to be significantly different between these two strains. Acetylcholine and nitroprusside were as effective in PAR2-HET as in PAR2-WT, which indicated that the lower PAR2-AP response in PAR2-HET was specific to PAR2 activation; and confirmed by finding that PAR2 agonist did not affect PAR2-KO. Ancillary to the functional differences between strains was a general observation that endothelium-dependent relaxations were larger in aortas from females than from males.

PAR2-HET have half the *par2* gene content of PAR2-WT, and yet the total PAR2 mRNA content in aortas of PAR2-HET was not different than in those of PAR2-WT. These data indicate that processes other than mRNA content alone regulate the constitutive PAR2 expression in aortas. The magnitude of attenuation of 2fly-induced relaxations in PAR2-HET vs. PAR2-WT was small, but consistent with subsequent results that attribute the differences between strains to a differential expression of spare receptors on the endothelium. According to the concept of spare receptors in receptor theory, the number of membrane surface receptors in cells exceeds the number required to elicit a maximal cellular response [15], [20]. Thus, the constitutive levels of PAR2 in PAR2-WT aortas may exceed that which is needed to be activated in order to observe the maximum effect when tested by the maximally effective concentrations of 2fly. In tissues of PAR2-HET, which are missing a *par*2 allele, the total number of PAR2 may be less than in PAR2-WT and yet, the number of receptors that would be bound to agonist could be only marginally reduced. Our spare receptor hypothesis was supported by an estimated four-fold lower *k*_GB-83_ in PAR2-HET compared to PAR2-WT, which indicates there are fewer spare receptors in PAR2-HET. The methods that we used to quantitatively estimate the spare receptors in aortas of PAR2-HET and PAR-WT are considered classical pharmacological approaches and have been used to examine other seven transmembrane G-protein coupled receptors [18], [21]. While the concept of spare receptors aligns with our observations and offers one explanation for the closer resemblance of PAR2-HET to PAR2-WT than to PAR2-KO, it does exclude other possible mechanisms. It is known that receptor number does not always correlate with biological responsiveness [22]. One could argue that PAR2-HET have less PAR2 expression than PAR2-WT and compensatory downstream mechanisms sustain the capacity to elicit a near maximal cellular response. Compensatory mechanisms have been reported in heterozygous null receptor mouse models, which resulted in apparently equivalent to wild-type phenotypes [13], [14]. Therefore, an increase in PAR2 signal transduction mediated at the level of G-protein activation in PAR2-HET could also explain why relaxations resulting from PAR2 activation in these animals more closely resembled PAR2-WT than PAR2-KO.

For more than a decade it has been reported that PAR2 activation of large caliber arteries follows a signal transduction mechanism like that of muscarinic receptor activation by acetylcholine. Similarly, there has been consistent interest in investigating the interaction of gender with vascular health. Particular attention has focused on estrogen and its actions on NO signal transduction [23]. Based on the experimental design of our study which requires numerous controls, it was possible for us to investigate whether the NO signal transduction system was altered in PAR2-HET, and whether there was any interaction between PAR2 and gender. In fact there were no differences between PAR2-HET and PAR2-WT for either acetylcholine- or nitroprusside-induced relaxations of aortas. These data indicate *par*2 heterozygosity did not affect NO signal transduction in the endothelium and smooth muscle of the aortas. Across PAR2 strains, acetylcholine-induced relaxations in females were larger than in males. We observed that these same sex-related differences for PAR2-mediated vasodilation in PAR2-WT and PAR2-HET. Surprisingly, nitroprusside-induced relaxations were larger in males than in females. In a recent study, it was reported that the PI3-kinase/endothelial NOS relaxation pathway of aortas in female type II diabetic mice was preserved whereas this pathway was attenuated in males [24]. This other study aligns with the findings in our current work, and highlights that non-sex related genotype mutations may produce *de novo* phenotypes that result in actual sex-dependent effects. Overall our approach to investigating the sex-related differences, which were relatively small was limited to a descriptive assessment between strains. In addition, our data reflects only the sex-related interactions in a healthy physiological model, so our results could underestimate the sex-related differences in response to pathological stresses. At the least, our data provides a starting point for planning in-depth examinations of sex-related differences in PAR2 vascular biology.

Considerable research has shown that heterozygous gene knockout cells do not necessarily have reduced mRNA transcript levels despite containing only one wild-type gene allele [25]–[27]. We have made an assumption that the results of the quantitative real-time PCR assay of aortas represents endothelial cell expression of PAR2, which was based on a wide review of literature describing PAR2 function in healthy mouse aortas. Though PAR2-HET inherit half of the genomic content of *par*2, PAR2 mRNA levels were not different than in PAR2-WT aortas. An intriguing hypothesis for future studies is that PAR2-HET cells at baseline conditions accumulate excess PAR2 mRNA and therefore, PAR2-HET produce enough PAR2 mRNA to match that in PAR2-WT. Likewise it is possible under the influence of a stimulus which initiates receptor turnover, PAR2-HET may not produce enough mRNA to meet the new protein synthesis demands [28]. In principal, both the mRNA turnover and the spare receptor hypothesis could be investigated further using immunological approaches to attempt to quantify receptor expression. Unfortunately, such a proposal regarding PAR2 is not without practical limitations. Our research group and others have published evidence that the current array of commercial available antibodies are ineffective for quantifying PAR2 by Western blot [6], [29]. Future development and optimization of immunocytochemistry techniques may eventually be used to further test these hypotheses. While it was beyond the scope of our study, it is interesting to consider whether the differences that we did observe between PAR2-HET and PAR2-WT would be magnified under *in vivo* conditions that stimulate PAR2 expression and activation, such as tissue inflammation.

In conclusion, cells containing only one PAR2 wild-type allele were unable to sustain the full wild type functionality of PAR2 in mouse aortas. PAR2-HET do not have the same PAR2 vascular responsiveness *in vitro* as PAR2-WT. These differences may be due to a reduced quantity of functional PAR2 in PAR2-HET versus PAR2-WT, despite equivalent mRNA content. Finally, relaxation of mouse aortas by PAR2-AP is higher in females than in males, which aligns with the broader literature on endothelial-dependent vasodilation and raises interesting questions about the possible interaction of gender with other PAR2-dependent activities. Therefore, future studies are warranted to investigate the specific mechanisms that lead to the differential effects of PAR2 agonist, PAR2 antagonist, spare receptor expression in PAR2-HET, and to evaluate mechanisms underlying the interaction of gender with PAR2 vascular activities.
